# The polyglutamine domain is the primary driver of seeding in huntingtin aggregation

**DOI:** 10.1371/journal.pone.0298323

**Published:** 2024-03-14

**Authors:** Adam Skeens, Chathuranga Siriwardhana, Sophia E. Massinople, Michelle M. Wunder, Zachary L. Ellis, Kaitlyn M. Keith, Tyler Girman, Shelli L. Frey, Justin Legleiter

**Affiliations:** 1 The C. Eugene Bennett Department of Chemistry, West Virginia University, Morgantown, West Virginia, United States of America; 2 The Department of Chemistry, Gettysburg College, Gettysburg, Pennsylvania, United States of America; 3 Rockefeller Neurosciences Institutes, West Virginia University, Morgantown, West Virginia, United States of America; 4 Department of Neuroscience, West Virginia University, Morgantown, West Virginia, United States of America; Rijksuniversiteit Groningen, NETHERLANDS

## Abstract

Huntington’s Disease (HD) is a fatal, neurodegenerative disease caused by aggregation of the huntingtin protein (htt) with an expanded polyglutamine (polyQ) domain into amyloid fibrils. Htt aggregation is modified by flanking sequences surrounding the polyQ domain as well as the binding of htt to lipid membranes. Upon fibrillization, htt fibrils are able to template the aggregation of monomers into fibrils in a phenomenon known as seeding, and this process appears to play a critical role in cell-to-cell spread of HD. Here, exposure of *C*. *elegans* expressing a nonpathogenic N-terminal htt fragment (15-repeat glutamine residues) to preformed htt-exon1 fibrils induced inclusion formation and resulted in decreased viability in a dose dependent manner, demonstrating that seeding can induce toxic aggregation of nonpathogenic forms of htt. To better understand this seeding process, the impact of flanking sequences adjacent to the polyQ stretch, polyQ length, and the presence of model lipid membranes on htt seeding was investigated. Htt seeding readily occurred across polyQ lengths and was independent of flanking sequence, suggesting that the structured polyQ domain within fibrils is the key contributor to the seeding phenomenon. However, the addition of lipid vesicles modified seeding efficiency in a manner suggesting that seeding primarily occurs in bulk solution and not at the membrane interface. In addition, fibrils formed in the presence of lipid membranes displayed similar seeding efficiencies. Collectively, this suggests that the polyQ domain that forms the amyloid fibril core is the main driver of seeding in htt aggregation.

## Introduction

HD is a fatal, neurodegenerative disease caused by the abnormal expansion of the CAG trinucleotide repeat encoding for the polyglutamine (polyQ) tract within the first exon of the huntingtin protein (htt) [1]. PolyQ expansion directly leads to the ordered aggregation of N-terminal fragments of htt into amyloid fibrils that coalesce into large inclusion bodies. Importantly, the rate of fibril formation directly correlates with polyQ length, with longer expansions enhancing aggregation [2, 3]. In addition to fibrils, htt forms a variety of complex oligomeric and amorphous aggregates both on and off pathway to fibril formation. Assigning a clear toxic role to the different aggregate forms remains elusive, but htt oligomers [4, 5], fibrils [6, 7], and inclusions [8, 9] all demonstrate some level of toxicity.

Amyloid aggregates, characterized by a distinct β-sheet rich fibrillar structure, represent a common feature of a variety of systemic and neurodegenerative diseases [10, 11]. Amyloid typically forms via a slow lag phase leading up to a nucleation event followed by fast elongation of fibrils [12]. However, pre-formed amyloid fibrils can act as nucleation centers that promote misfolding of native protein, accelerating fibril formation in a process termed seeding [13]. Seeding is enhanced by fibril fragmentation, which creates additional active ends [14, 15]. In addition, seeds can be taken up by adjacent cells where they induce aggregation, amplifying and spreading disease. In particular, intercellular seeding is implicated in the spread of HD pathology [16–18]. Seeding of htt aggregation across neurons occurs via tunneling nanotubes [19] and synaptic transfer release of exosomes [20], which allows for non-contact transmission between neurons and is a hallmark of disease progression.

Within HD, the seeding phenomenon may be complicated by additional factors associated with htt aggregation, i.e. polyQ length. While the polyQ domain remains the primary driver of aggregation, adjacent flanking sequences modify the process [21]. The polyproline (polyP) rich domain (PRD) directly following the polyQ domain slows fibrillization [22, 23] but does not impact the overall fibril core structure [24]. The first seventeen N-terminal amino acids (Nt17) directly preceding the polyQ domain promote aggregation [23]. Nt17 facilitates the formation of α-helix rich oligomers that promote nucleation by bringing polyQ stretches into close proximity [25]. In terms of fibril structure, distinct fibrils (toxic vs nontoxic) result from incubating htt-exon1 at different temperatures (4 vs 37°C respectively) [26]. Structural differences between these two fibril forms are isolated to the PRD, with no observable changes in the Nt17 or polyQ domains within the fibril [27]. Importantly, different fibril types (formed under unique conditions) displayed varying abilities to seed aggregation, with the ability to seed aggregation correlating with toxicity. In addition to the effect of the flanking sequences, HD is an autosomal dominant disease, meaning only one expanded allele is required for pathogenesis. Heterozygous and homozygous brains of HD patients have the same age of onset suggesting the presence of htt with unexpanded CAG repeat length plays a minimal role in disease onset [28, 29]. Htt fibrils recruit wild type htt into their structure through cross-seeding of polyQ lengths both *in vitro* and in cellular environments [30, 31]. The wild type htt fibrils maintain the same fibril structure as the templating aggregate but with a less extended polyQ core.

Due to its ability to form an amphipathic α-helix, Nt17 also promotes the association of htt with lipid membranes [32]. Importantly, the interaction with lipid membranes further modifies aggregation, and thus may play a role in seeding aggregation. For example, POPC/POPS vesicles enhance fibril formation by promoting a unique aggregation pathway, but ultimately leads to similarly structured fibrils [33]. More generally, anionic lipid headgroups tend to enhance htt fibrillization in comparison to zwitterionic head groups [34]. The association between Nt17 and simple model membranes is influenced by complementarity between hydrophobic residues and membrane defects, which alters the ease by which Nt17 can partition into the bilayer [35]. More complex systems (mitochondrial membrane mimics, brain and tissue lipid extracts) often reduce fibril formation compared to htt aggregation in the absence of lipid [36–38]. However, little is known with regard to how membranes influence seed formation or even the seeding process.

Htt aggregates transfer from cell-to-cell, seeding aggregation in the invaded cell and impacting disease progression [16–18]. Due to this, understanding the role of protein sequence and cellular environment on the seeding process can provide insight into HD. Here, we demonstrate that the seeding process enhances toxicity in a *C*. *elegans* strain expressing nonpathogenic htt. With flanking sequences being well established as modifiers of htt aggregation, we investigated if these regions also modified seeding efficiency of polyQ fibrils by using a variety of synthetic peptides containing different flanking sequences to produce seeds. The impact of lipid interfaces on seed formation and on the seeding process was also investigated. Collectively, these studies point to a primary role of the polyQ domain, which makes up the central fibril core, in the seeding phenomenon.

## Materials and methods

### Expression and purification of (GST)-htt-exon1 fusion protein

Both (GST)-htt-exon1(46Q) and (GST)-htt-exon1(20Q) were purified as described previously [39]. In short, both proteins were expressed in E. coli. Expression was induced with isopropyl β-D-thiogalactoside for 4 h at 37°C. The cells were lysed with lysozyme (0.5 mg/mL) and probe sonicated. The lysates of each construct were purified with separate GST affinity columns using liquid chromatography (BioLogic LPLC, BioRad) to avoid potential cross contamination. Relevant fractions were collected, verified by SDS-PAGE, and placed in dialysis for 48 h against Tris buffer (50 mM Tris-HCl, 150 mM NaCl, pH 7.4). Protein concentrations were determined by Bradford assays. Prior to each experiment, pre-existing aggregates were removed via centrifugation (20,000 ×g at 4°C for 45 min). Factor Xa protease (New England Bio Labs) was used to cleave the GST tag and initiate aggregation.

### Htt peptide preparation

Each peptide was dissolved in a 1:1 mixture of hexafluoroisoproponal and trifluoroacetic acid for 12 h without agitation. Solvent was evaporated with the use of a vacufuge. The peptide films were dissolved in DMSO to a concentration of 2 mM and kept at 4°C until needed. For the production of the seeding species, each peptide was diluted in Tris buffer to a concentration of 200 uM and allowed to aggregate at 37°C until fibrils formed. After the required incubation period, fibrils of each peptide were bath sonicated for 1 h to induce fragmentation.

### Lipid vesicle preparation

Total brain lipid extract (TBLE, Avanti Polar Lipids) was hydrated in 50 mM Tris buffered saline at 60°C to suspend lipids in solution. The hydrated TBLE was subject to 10 freeze/thaw cycles in liquid nitrogen, followed by bath sonication for 15 min to promote vesicle formation.

### *C*. *elegans* culture and fluorescence imaging

A control *C*. *elegans* strain (N2) and strains expressing htt-513 with either a nonpathogenic (Q15, EAK102) or pathogenic (Q128, EAK103) were purchased from the Caenorhabditis Genetic Center and maintained at 20°C under standard protocols [40]. Age synchronized worms were obtained using previously published methods [40]. For viability assays, day 1 adult worms of both N2 and EAK102 were exposed to various concentrations of 1 min probe sonicated htt-exon1(46Q) seeds in a liquid culture medium in a 96 well plate format and maintained at 20°C. Each well contained 10–15 worms, and each condition was replicated in a total of 9 wells (3 independent plates with 3 wells per condition on each plate). For experiments with peptide-derived seeds, both N2 and EAK102 worms were exposed to 5 μM of each seed, and each condition was replicated in a total of 6 wells (2 independent plates with 3 wells per condition on each plate). OP50 concentration in the medium was set to 0.05 mg/mL. For fluorescence microscopy (Olympus Spinning Disc Fluorescent Confocal Microscope), day 1 adult worms of EAK102 were exposed to 10 μM htt-exon1(46Q) seeds or 5 μM peptide seeds in a liquid culture medium at 20°C. For comparison, day 1 adult worms of EAK102 and EAK103 were maintained without exposure to any seeds. After 24 h of exposure, worms were removed from the culture medium and were suspended in 15 mg/mL 2,3-Butanedione monoxime (BDM) for 30 minutes to paralyze. The number of inclusions per worm was measured from fluorescence images using the image processing toolbox in Matlab.

### Thioflavin T (ThT) assay

Each htt-exon1 (46Q or 20Q) protein was diluted to a final concentration of 20 uM in the presence of 40 μg/mL ThT (Sigma-Aldrich). The aggregation of htt-exon1 in the presence of each peptide seed was assayed in a 96-well plate (100 uL per well) on a microplate reader (Molecular Devices, SpectraMax M2) at 37°C for 18 h, reading every 10 min with a 440 nm excitation and 484 nm emission. ThT assays with lipid had a 20:1 lipid:protein ratio.

### Polydiacetylene (PDA) vesicle binding assay

TBLE-PDA vesicles were prepared as described [41]. In brief, 10,12-triosadiynoic acid (GFS Chemicals) and TBLE (Avanti lipids) were dissolved at a 2:3 molar ratio in a solution of 1:4 ethanol:choloroform. Excess solvent was evaporated with a stream of N2 and resuspended in 50 mM Tris buffered saline heated to 70°C. Probe sonication 10 min was performed to achieve an opaque solution. The resulting solution was stored at 4°C overnight for self-assembly of the TBLE-PDA vesicles. The TBLE-PDA vesicles were polymerized through exposure to 254 nm illumination for 10 minutes at room temperature with stirring, resulting in a blue solution. The assay was performed in a clear 96-well plate using a microplate reader (Molecular Devices, SpectraMax M2) at a final htt-exon1(46Q) concentration of 20 μM at 25°C and included a positive NaOH control (100 μM) and a negative 50 mM Tris buffered saline control. The colorimetric response (CR) was determined every 10 minutes by measuring blue and red light absorbance at 640 nm and 500 nm) and using:

$$CR=\frac{PB_{0}-{PB}_{1}}{{PB}_{0}}$$ (1)

where PB is defined as A_640nm_/(A_640nm_+ A_500nm_). PB_0_ is determined from the negative control of Tris buffered saline and PB_1_ is determined from each condition.

### Atomic Force Microscopy (AFM)

For ex situ AFM experiments, the appropriate htt-exon1 construct (20 uM) was incubated in the presence or absence of each peptide seed at 37°C and 1400 rpm in an orbital thermomixer for 8 h. After the required incubation time, 5 uL aliquots of each sample were deposited onto freshly cleaved mica (Ted Pella Inc.) for 1 min, washed with 200 uL 18 MΩ·cm ultrapure water, and dried with a stream of filtered air. All samples were imaged using a Nanoscope V MultiMode atomic force microscope (VEECO, Santa Barbara, CA) equipped with a closed loop vertical engage J-scanner and silicon-oxide cantilevers with a resonance frequency of ~300 kHz and nominal spring constant of 40 N/m. All images were analyzed using MATLAB equipped with the image processing toolbox (MathWorks) as described [42].

### Circular Dichroism (CD) spectroscopy

Htt-exon1(46Q) (20 μM) was incubated without and with peptide derived seeds for 24 h. Samples were diluted to 10 μM in Tris buffer and 10 μM GST was run as a control. CD spectra were collected on a JASCO J-1500 spectropolarimeter using a 1 mm pathlength quartz cuvette. Spectra are baseline corrected and averaged over three scans, measured from 260 nm to 190 nm with a 0.2 nm data interval, and 4 s data averaging.

## Results

### Seeding occurs across polyQ lengths in *C*. *elegans*

**Fig 1:**
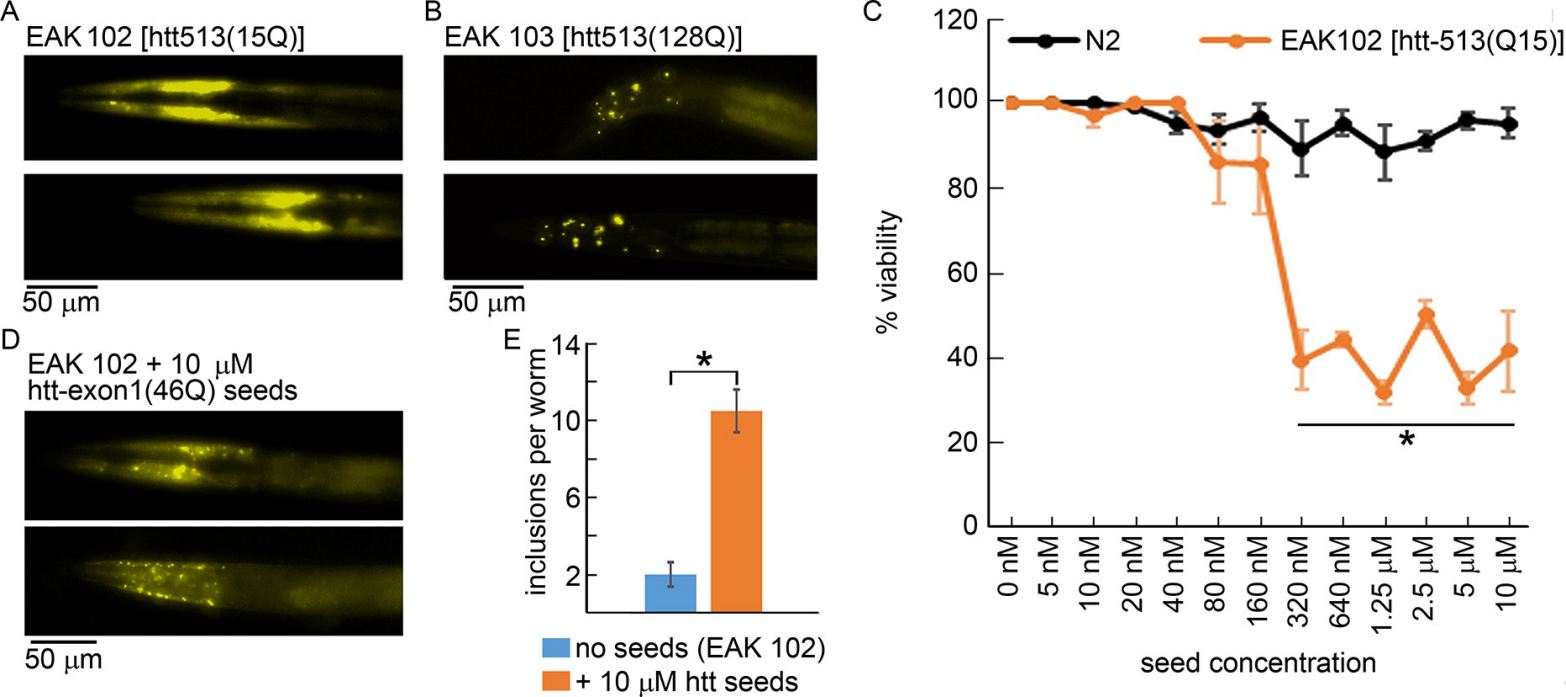
Htt-exon1(46Q) fibrils seed aggregation in a *C*. *elegans* strain (EAK102) expressing a non-pathogenic N-terminal htt fragment [htt-513(Q15)]. Representative fluorescence microscopy images of the (A) EAK102 and (B) EAK103 *C*. *elegans* strains that express htt513(15Q) and htt513(128Q), respectively. (C) Worm viability of EAK102 and a control strain (N2) 48 h after exposure to various concentrations of htt-exon1(46Q) seeds. Error bars represents standard error of the mean. * represents p < 0.01 based on a T-test comparing N2 and EAK102 worm viability at a given dose (n = 3 for both conditions). (D) Representative fluorescence microscopy images and (E) quantification of visible inclusion bodies per worm comparing control EAK102 worms to EAK102 worms exposed to 10 μM htt-exon1(46Q) seeds. Error bars represent standard error of the mean. Error bars represents standard error of the mean. * represents p < 0.01 based on a T-test comparing the number of inclusions observed in EAK102 worms to those treated with 10 μM htt seeds (n = 5 for the no seeds condition and n = 8 for the seeded condition).

To demonstrate seeding across polyQ lengths and htt fragments in a living system, the ability of fibrils derived from incubations of a GST-htt-exon1 fusion protein with 46 repeat glutamine residues (htt-exon1(46Q)) to seed aggregation in a *C*. *elegans* strain (EAK102) expressing a YFP-labeled N-terminal htt fragment containing the first 513 residues with a nonpathogenic length polyQ domain (15 residues long) [43] was determined. Beyond the mismatch in polyQ length between the htt-exon1(46Q) used to form fibrils and the htt fragment expressed in the worms, Htt-513 is also slightly longer than exon1. Expression of this htt-513(15Q) in *C*. *elegans* does not induce any phenotype, and the htt remains predominately diffusely distributed [43]. That is, no or few inclusions are observed (Fig 1A). For comparison, increasing the polyQ domain length of htt-513 to 128Q in a *C*. *elegans* strain (EAK103) induces a thrashing deficit, reduces viability, and invokes the formation of visible htt inclusions [43] (Fig 1B). Inclusion formation in the EAK103 worms expressing htt-513(128Q) is indicative of pathological aggregation.

To create seeds, purified htt-exon1(46Q) was aggregated to obtain a large population of fibrils (verified via AFM imaging). Htt-exon1(46Q) fibrils were sonicated to increase the efficiency of uptake and seeding. Control wild type N2 worms that do not express any htt fragments and the EAK102 worms expressing htt-513(Q15) were exposed to these fibril seeds at concentrations ranging from 5 nM to 10 μM, and the viability of the worms was determined 48 h after exposure (Fig 1C). At all concentrations, the viability of N2 worms were relatively unaffected by exposure to htt fibrils (minimum viability for any condition was 88%), suggesting that preformed fibrils were not toxic to worms. However, EAK102 worms expressing nonpathogenic htt-513(15Q) were sensitive to htt-exon1(46Q) fibrils seeds at concentrations as low as 320nM as viability dropped to ~40%, a statistically significant (p < 0.01) decrease compared with control at a given dose of seeds. In addition, exposure to htt-exon1(46Q) seeds invoked the formation of a significant number of visible inclusions in EAK102 worms expressing htt-513(Q15) (Fig 1A, 1D and 1E), reminiscent of those observed in EAK103 worms. Importantly, this demonstrates that htt fibrils are readily taken up by worms, that seeding occurred across polyQ lengths, that htt fragments with nonpathogenic polyQ domains are susceptible to seeding, and seeded aggregation leads to toxicity.

### Seeding of htt is independent of flanking sequence

**Fig 2:**
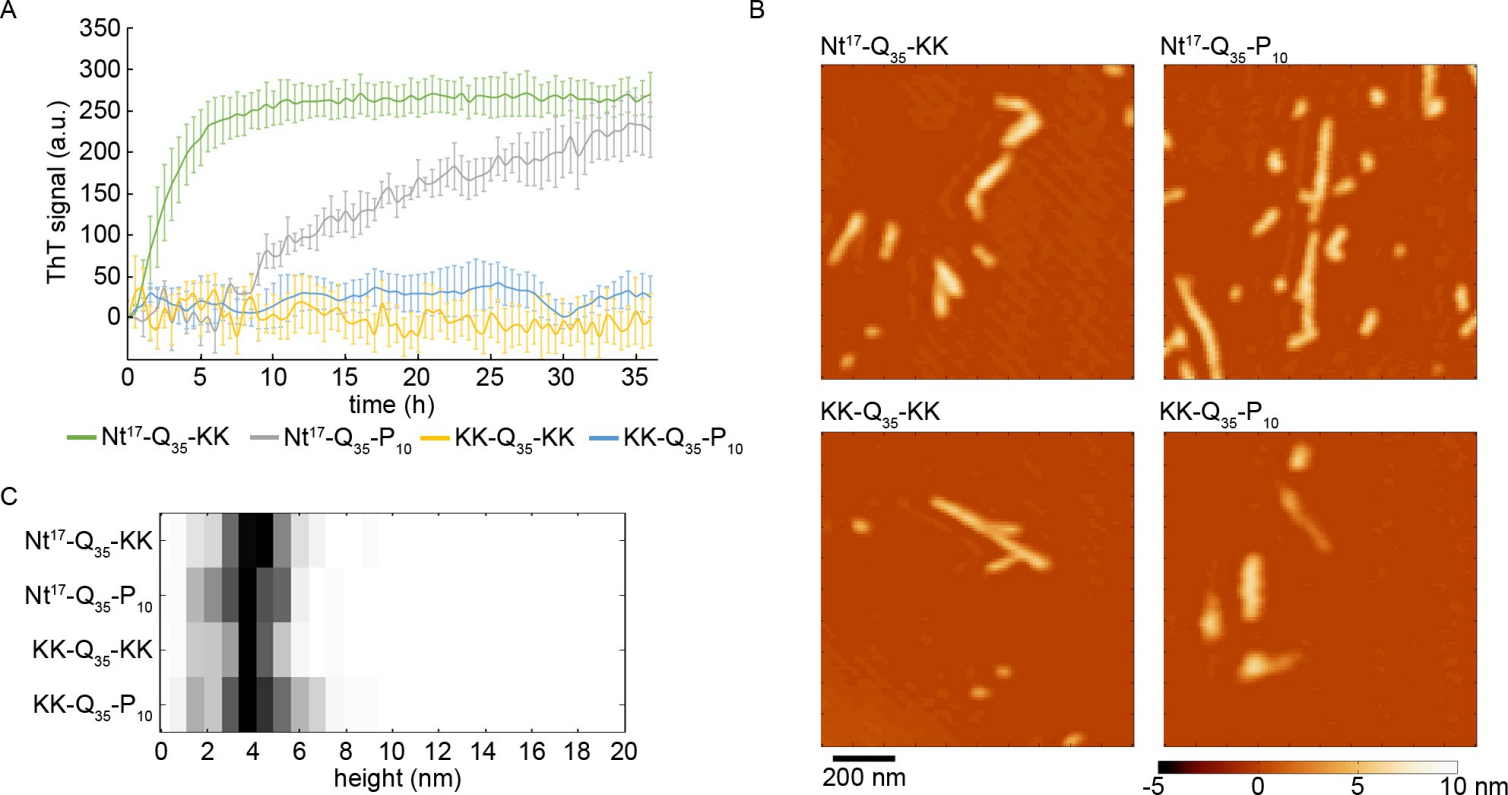
Fibrils formed by htt-mimicking peptides. (A) ThT assay demonstrating the different rate of fibril formation for the Nt^17^-Q_35_-KK, Nt^17^-Q_35_-P_10_, KK-Q_35_-KK, and KK-Q_35_-P_10_ peptides. Error bars represent standard error of the mean (n = 3 for all conditions). (B) Representative *ex situ* AFM images of fibrils formed from Nt^17^-Q_35_-KK, Nt^17^-Q_35_-P_10_, KK-Q_35_-KK, and KK-Q_35_-P_10_. (C) Histograms of the average height along the contour of fibrils corresponding to each htt peptide.

With the demonstration of seeding of nonpathogenic htt fragments in *C*. *elegans*, htt seeding mechanisms were further explored *in vitro*. With flanking sequence surrounding the polyQ domain readily influencing htt aggregation [22, 23], we postulated that these flanking regions may also influence seeding efficiency. To determine if flanking sequences influence seeding of htt fibrillization, synthetic peptides with varying flanking sequences were incubated to obtain seed-competent fibrils. These peptides include KK-Q_35_-KK, Nt^17^-Q_35_-KK, KK-Q_35_-P_10_-KK, and Nt^17^-Q_35_-P_10_-KK. While each of these peptides have the same length polyQ domain, they have different combinations of the Nt17 and polyP flanking sequences. Lysine residues at the different termini were included to aid in solubility of some synthetic peptides [44]. Varying incubations times were required to obtain seed-competent fibrils, as each peptide aggregates at varying rates [23]. Importantly, the time for each peptide to aggregate was consistent with previous reports [23]. That is, Nt^17^-Q_35_-KK and Nt^17^-Q_35_-P_10_-KK aggregated within a few hours based on ThT assays (Fig 2A) whereas, KK-Q_35_-KK and KK-Q_35_-P_10_-KK did not invoke a ThT signal within 36 h. Typically, 9–14 days were required for KK-Q_35_-KK and KK-Q_35_-P_10_-KK to significantly fibrillize. Due to these varying rates, atomic force microscopy (AFM) was used to monitor each peptide incubation for fibril formation (Fig 2B).

Considering that distinct fibril polymorphs of htt-exon1 previously displayed varying seeding efficiencies [27], analysis of the resulting fibril morphology derived from each peptide was performed. While various incubation times were required to obtain fibrils for each peptide, the morphology of the fibrils were remarkably similar (Fig 2B). As the thickness of fibrils, measured by the height in AFM images, can vary along the contour of the aggregate, the average height along the contour of individual fibrils was determined (Fig 2C). For each peptide the mode thickness of fibrils was ~4 nm. While AFM data alone does not confirm similar structure, the similarity in morphology is suggestive that each polyQ synthetic peptide formed similar fibril cores. Importantly, though polyQ-based fibrils display intrinsic polymorphism along the polyQ fibrils core [45], the polymorphism in htt fibrils is typically associated with the PRD [27]. In the peptides used here, the PRD, which contains 50 residues in htt-exon1, has been truncated to only contain a 10-residue long polyP sequence.

**Fig 3:**
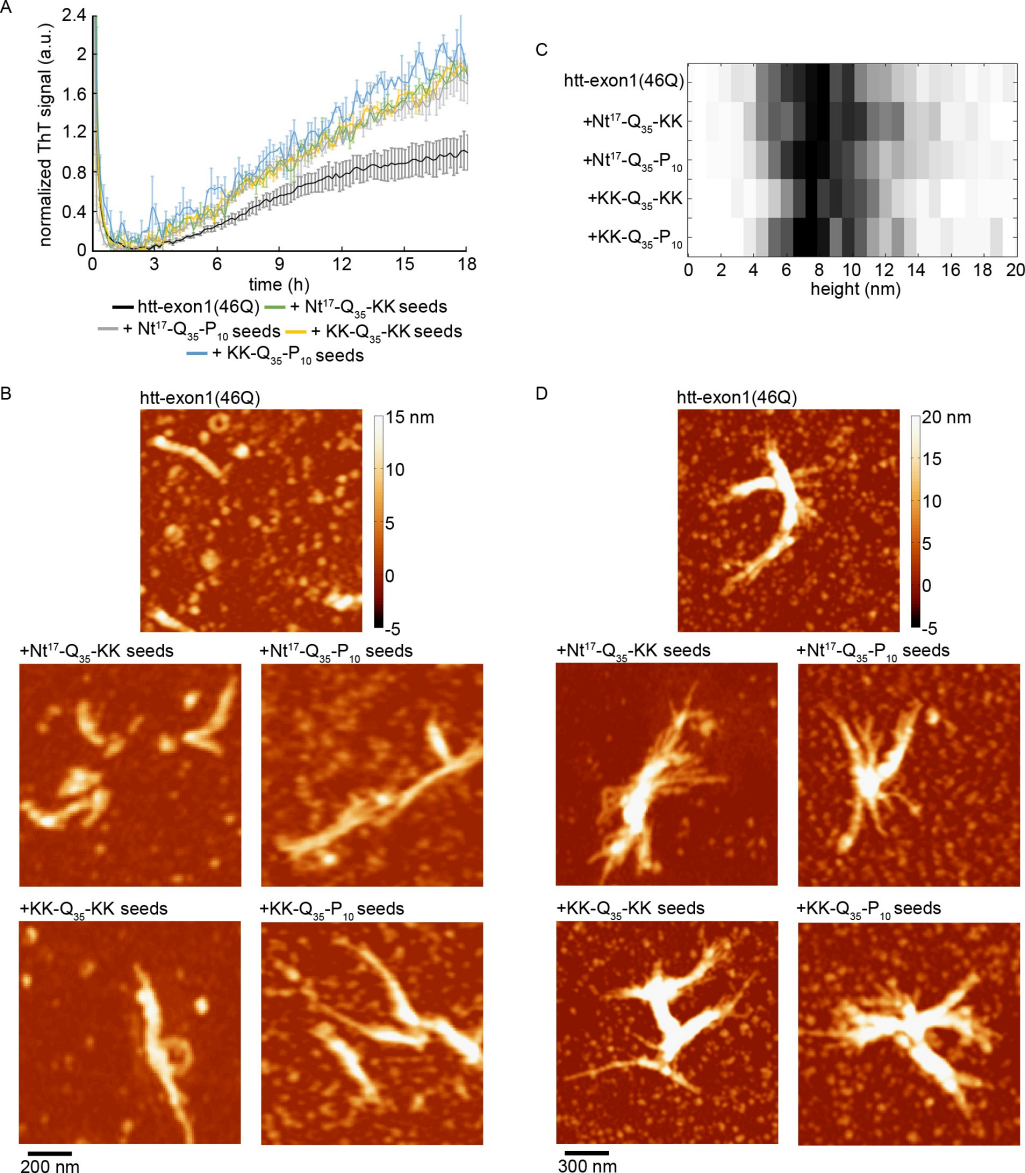
The impact of peptide seeds on htt-exon1(46Q) aggregation. (A) ThT assays tracking the impact of seeds comprised of Nt^17^-Q_35_-KK, Nt^17^-Q_35_-P_10_, KK-Q_35_-KK, or KK-Q_35_-P_10_ on htt-exon1(46Q) aggregation. The fluorescence signals were normalized to the control htt-exon1(46Q) alone aggregation reaction. Error bars represent standard error of the mean (n = 3 for all conditions). (B) Representative *ex situ* AFM images comparing fibrils of htt-exon1(46Q) formed in the absence and presence of seeds comprised of Nt^17^-Q_35_-KK, Nt^17^-Q_35_-P_10_, KK-Q_35_-KK, or KK-Q_35_-P_10_. (C) Histograms of the average height along the contour of htt-exon1(46Q) fibril formed in the absence or presence of the indicated peptide seeds. (D) Representative *ex situ* AFM images of htt-exon1(46Q) fibril bundles aggregated in the absence and presence of seeds comprised of Nt^17^-Q_35_-KK, Nt^17^-Q_35_-P_10_, KK-Q_35_-KK, or KK-Q_35_-P_10_.

After probe sonicating fibrils to enhance seeding efficiency, seeds derived from the different peptides were incubated with htt-exon1(46Q)). Cleaving of the GST tag by addition of Factor Xa initiates aggregation, and fibril formation was measured using a ThT assay at 20 μM htt-exon1(46Q) and 125 nM seeds where protein concentration was determined by Bradford assays (Fig 3A). Under these conditions, seeds derived from all four peptides accelerated aggregation to the same degree (by approximately a factor of 2 after 18 h compared to the htt alone control), suggesting that the polyQ plays the primary role in htt seeding independent of flanking sequences. The increased ThT signal at 18 h associated with each peptide-derived seed was significantly (P > 0.05) larger compared to the control htt-exon1(46Q) incubation without any seeds. However, none of the ThT signals for seeded experiments were statistically different from each other.

Having determined that all four peptides seeded htt aggregation to the same extent, AFM was used to characterize the impact of the different seeds on the resulting htt-exon1(46Q) fibrils (Fig 3B–3D). Using the same conditions as the ThT assays, each seeding incubation was aliquoted onto mica for analysis after 18 h of incubation. For htt-exon1(46Q) deposited on mica, individual fibrils and fibril bundles were observed. Morphologically similar fibrils (Fig 3B) and fibril bundles (Fig 3D) appeared in the presence of seeds derived from each peptide. Again, the average height along the fibril contour was determined for each condition (Fig 3C). As fibril bundling arbitrarily increases measured height and obscures the contour of individual fibrils, only fibrils protruding from fibrils were included in this analysis. The seeded and unseeded htt-exon1(46Q) fibrils had similar thickness distributions (mode average height along the contour of ~7–8 nm), indicating that the underlying structure of the fibrils were likely similar across all conditions, supporting a primary role in fibril formation for the polyQ domain, which makes up the core of fibrils based on solid state NMR structures [45, 46]. The larger height along the contour of these seeded fibrils compared to the peptide fibrils used as seeds may reflect the larger polyQ domain in htt-exon1(46Q).

**Fig 4:**
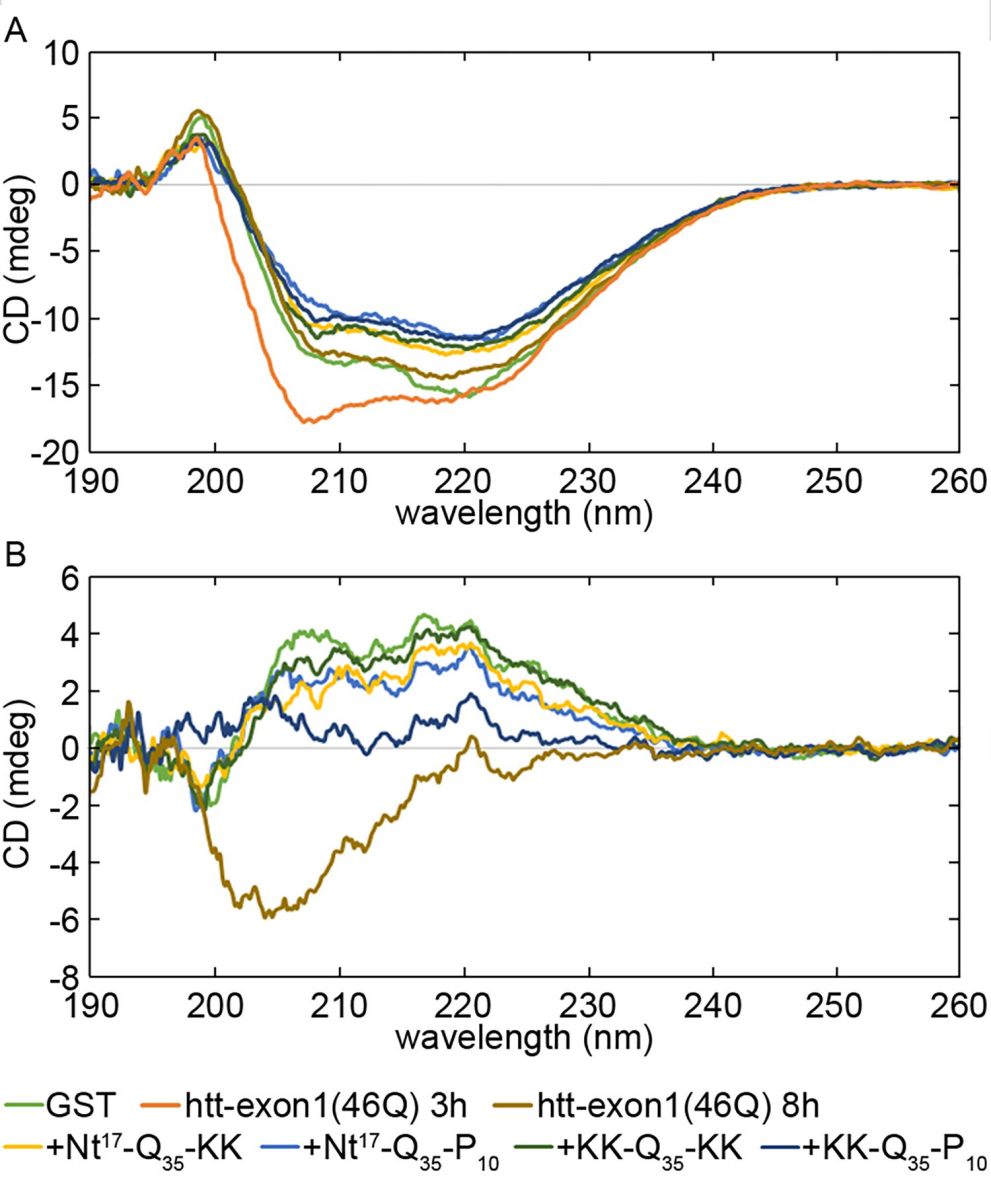
CD analysis of resulting fibril structure. (A) CD spectra of htt-exon1(46Q) aggregates formed in the absence (htt-exon1(46Q) 8h) or presence of seeds derived from Nt^17^-Q_35_-KK, Nt^17^-Q_35_-P_10_, KK-Q_35_-KK, or KK-Q_35_-P_10_ peptides. For comparison, a spectra of htt-exon1(46Q) after 3 h of incubation without seeds is provided, which is a condition comprised predominately of oligomers. Due to the presence of GST, a GST spectra is also provided. (B) CD spectra of htt-exon1(46Q) with the subtraction of the GST spectra.

To further test if the resulting htt-exon1(46Q) fibrils were structurally similar, CD spectra were obtained of the fibrils formed with and without seeds after 24 h of incubation (Fig 4A). While there is a large background from GST in our experimental system, all of the fibrils formed in the presence of peptide-derived seeds had similar CD profiles that were distinct from GST spectra as shown in the difference spectra where the GST signal is subtracted (Fig 4B). More specifically, the predominant features in the GST spectra indicate α-helical character with negative intensity peaks at 208 and 222nm while the fibril spectra are flattened in that region due to an increase in negative CD intensity at 217nm, likely stemming from increased β-sheet contribution from the fibril species. In addition, the spectra of htt-exon1(46Q) formed in the absence of any seeds was most similar to GST, suggesting less overall β-sheet content in that condition which is consistent with the smaller signal increase in the ThT assays. Furthermore, CD spectra of htt-exon1(46Q) after 3 h of incubation (a time point dominated by oligomers) is clearly different compared to those of htt fibrils with increased negative intensity at 208 and 222 nm stemming from the relatively rich α-helical content of htt oligomers [47]. This apparent similarity in seeded fibril structure, as evidenced by comparable CD spectra, is consistent with the intrinsic polymorphism observed within htt fibrils [41].

### Seeding occurs across glutamine length independent of flanking sequence

**Fig 5:**
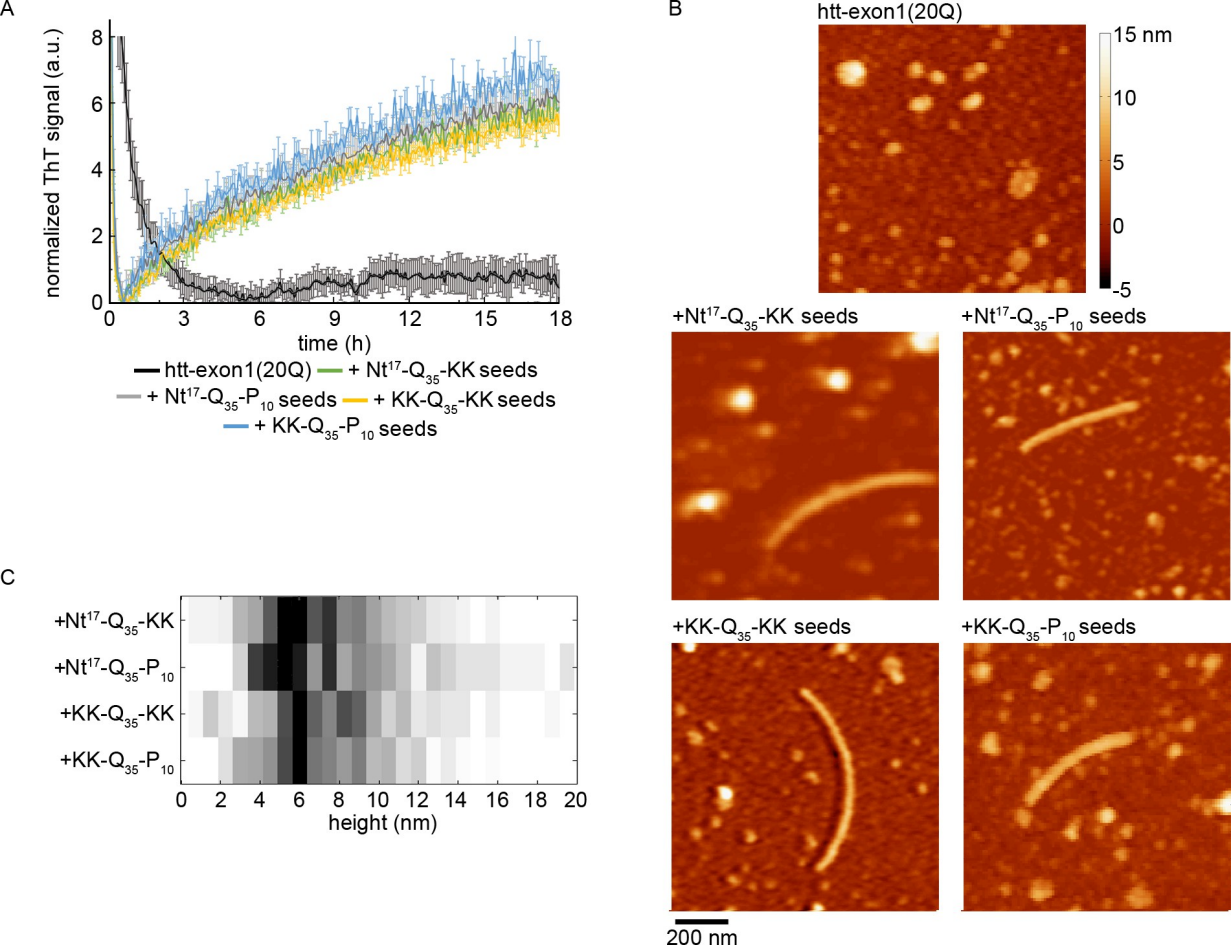
The impact of peptide seeds on htt-exon1(20Q) aggregation. (A) ThT assays tracking the impact of seeds comprised of Nt^17^-Q_35_-KK, Nt^17^-Q_35_-P_10_, KK-Q_35_-KK, or KK-Q_35_-P_10_ on htt-exon1(20Q) aggregation. The fluorescence signals were normalized to the control htt-exon1(20Q) alone aggregation reaction. Error bars represent standard deviation (n = 3 for all conditions). (B) Representative *ex situ* AFM images comparing fibrils of htt-exon1(20Q) formed in the absence and presence of seeds comprised of Nt^17^-Q_35_-KK, Nt^17^-Q_35_-P_10_, KK-Q_35_-KK, or KK-Q_35_-P_10_. Colored lines in each image correspond to the height profiles. (C) Histograms of the average height along the contour of htt-exon1(20Q) fibril formed in the presence of the indicated peptide seeds. A distribution for htt-exon1(20Q) fibrils formed in the absence of seeds is not provided because no fibrils were observed under that condition.

Having observed that seeding occurs independent of flanking sequence with pathogenic length polyQ domains, investigations then aimed to determine the ability of the different peptide-derived seeds to induce fibrillization in nonpathogenic length htt were pursued. With the same conditions as used for investigations of htt-exon1(46Q), ThT assays and AFM analysis were performed on various seeds incubated with GST-htt-exon1 fusion protein with 20 glutamine repeats (htt-exon1(20Q), Fig 5A). Similar to results with pathogenic htt-exon1, all four peptide-derived seeds induced fibrillization of htt-exon1(20Q), even though htt-exon1(20Q) did not form fibrils in the absence of seeds. All four peptide-derived seeds enhanced fibrillization to the same extent, with a resulting ThT signal ~8-fold higher than the signal for control htt-exon1(20Q), which was statistically significant (p < 0.005). However, the maximum ThT signal associated with each seeded incubation were not significantly different from each other. To investigate aggregate morphology, each seeding incubation was aliquoted onto mica for analysis after 18 h of incubation. With the control incubation of htt-exon1(20Q) alone, no fibrils were observed by AFM, but oligomers of various sizes were present (Fig 5B). In contrast, fibrils were present in htt-exon1(20Q) incubations with each of the peptide-derived seeds. Based on the distribution of average height along the fibril contour (Fig 5C), the morphology of these fibrils was similar under all seeding conditions with a thickness of ~6 nm. The thickness of htt-exon1(20Q) fibrils was smaller than the htt-exon1(46Q) fibrils, which may be due the shorter polyQ domains. The htt-exon1(20Q) fibrils were thicker than the polyQ-peptide seed fibrils (Fig 2C), but it is important to recall that the none of the peptides contain a full PRD. Collectively, these results reinforce the importance of the polyQ domain within seeding-competent fibrils.

### Htt peptide seeds alter aggregation in the presence of lipids

**Fig 6:**
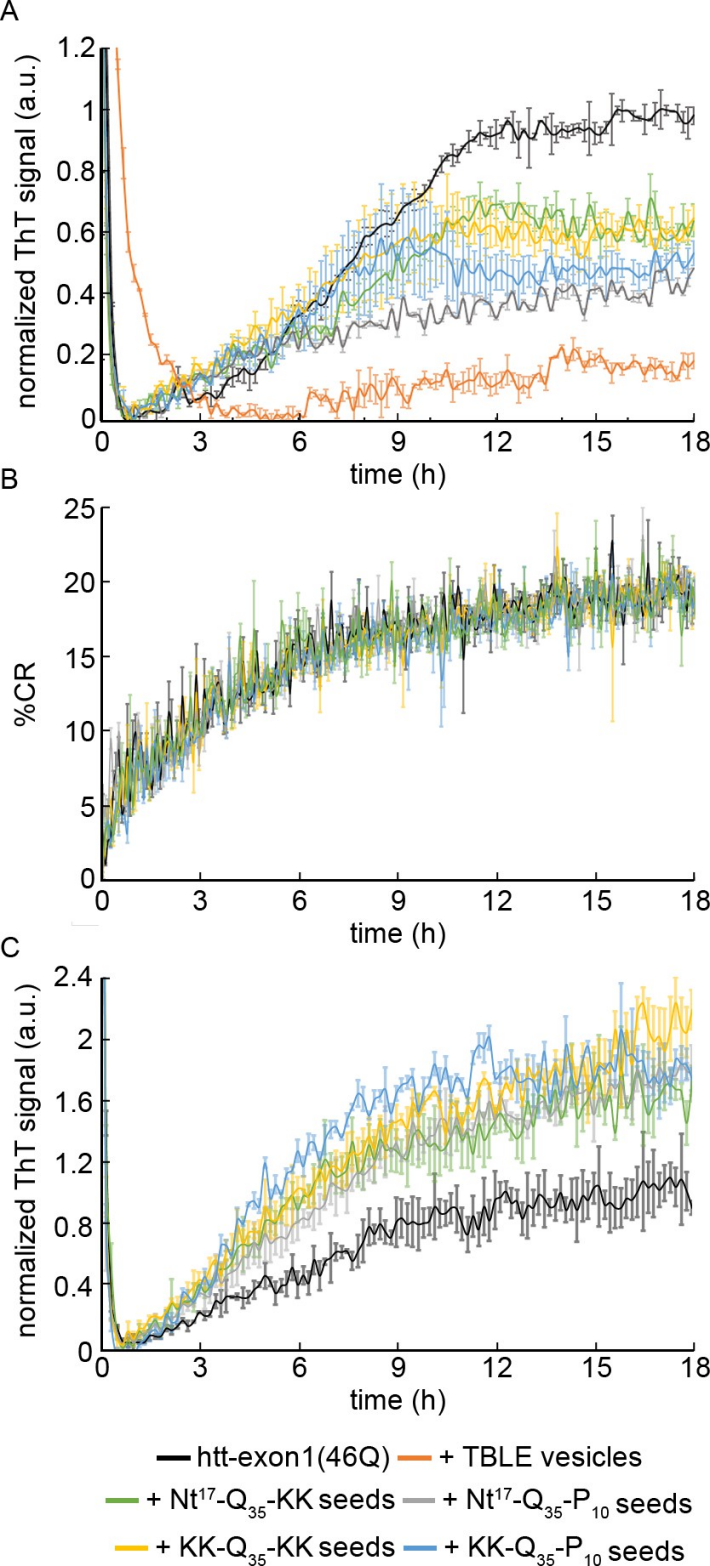
The impact of lipids on the seeding of htt-exon1(46Q) by peptides. (A) ThT assays tracking the impact of TBLE vesicles (20:1 lipid:protein molar ratio) on the ability of seeds comprised of Nt^17^-Q_35_-KK, Nt^17^-Q_35_-P_10_, KK-Q_35_-KK, or KK-Q_35_-P_10_ to alter htt-exon1(46Q) aggregation. (B) PDA/TBLE assay measuring the impact of seeds comprised of Nt^17^-Q_35_-KK, Nt^17^-Q_35_-P_10_, KK-Q_35_-KK, or KK-Q_35_-P_10_ on the ability of htt-exon1(46Q) to bind TBLE vesicles. (C) Htt-exon1(46Q) aggregation tracked by ThT assay in the presence of Nt^17^-Q_35_-KK, Nt^17^-Q_35_-P_10_, KK-Q_35_-KK, or KK-Q_35_-P_10_ seeds produced in the presence of POPG vesicles. All ThT plots were normalized to the control htt-exon1(46Q) alone aggregation reaction. Error bars represent standard deviation for each plot (n = 3 for all conditions).

As the presence of lipid membranes invoke a unique fibrillization pathway that is mediated by the Nt17 flanking sequence [33], the fibrils formed by the four peptides were tested for their ability to seed htt-exon1(46Q) aggregation in the presence of total brain lipid extract (TBLE) vesicles using ThT assays (Fig 6A). TBLE vesicles were chosen due to their physiologically relevant mix of lipid components. In addition, the lipid to htt-exon1(46Q) molar ratio was 20:1. At this ratio, TBLE vesicles are known to slow htt fibril formation [38, 48], which was recapitulated here. This provides a second rationale for using TBLE vesicles, as we avoided lipid systems for this assay that already accelerate htt aggregation. In the presence of vesicles alone, htt-exon1(46Q) caused a significant (p < 0.0001, t-test) reduction in aggregation with an 82% reduction in fibril formation relative to htt in the absence of lipids. With the introduction of seeds derived from each peptide to htt-exon1(46Q) incubations with lipid vesicles, fibrillization was significantly (p < 0.01, t-test) enhanced relative to htt-exon1(46Q) incubated in the presence of lipids; however, the overall signal did not reach the level associated with htt-exon1(46Q) incubated in the absence of lipids (Fig 6A, p < 0.01). Overall, the different peptide-derived seeds increased aggregation about 2.5-fold relative to htt-exon1(46Q) aggregation without seeds in the presence of TBLE vesicles, and there was not a statistical difference in the seeding potential of the different peptide-derived seeds.

Next, a colorimetric polydiacetylene (PDA)/lipid vesicle binding assay was performed to determine if the different seeds impacted the ability of htt-exon1(46Q) to interact with lipid interfaces (Fig 6B). The PDA/lipid vesicles exhibit a measurable colorimetric response (CR) upon binding of molecules and peptides to the vesicle. Based on this assay, introduction of any of the peptide-derived seeds had no impact on the ability of htt-exon1(46Q) to bind lipid vesicles. Together, the ThT and PDA assays suggest that the increase in aggregation in the presence of seeds results from the fraction of htt-exon1(46Q) not bound to the lipid vesicle. That is, seeding primarily targets lipid-free htt-exon1(46Q). Some sequestration of htt-exon1(46Q) to the membrane surface reduces the available htt-exon1(46Q) that can be involved in seeded aggregation, resulting in a ThT signal between the htt-exon1(46Q) alone conditions in the presence and absence of TBLE vesicles.

Aggregation is altered in the presence of lipids in a flanking sequence dependent manner [32]. This unique aggregation on lipid interfaces could alter fibril structure and their ability to seed fibril formation. To determine if the seeding efficacy of fibrils formed in the presence of lipid interfaces was dependent on flanking sequences, the ability of fibrils of the four peptides grown in the presence of lipids to seed aggregation was tested. As POPG accelerates htt aggregation [34, 49], the four peptides were incubated with POPG vesicles in a 30:1 lipid to peptide ratio to obtain fibrils. Fibril formation was verified via AFM with no observable difference in fibril morphology. The fibrils from all four peptides were sonicated and used to seed (125 nM) htt-exon1(46Q) (20 μM) aggregation (Fig 6C). Again, all four of the peptide-derived seeds enhanced fibrillization to similar levels (~1.8-2-fold increase in ThT signal compared to control, p < 0.01), again suggesting that the polyQ domain plays the primary role in seeding htt aggregation even with aggregated formed in the presence of lipids.

### Peptide-derived seeds are toxic to *C*. *elegans* expressing nonpathogenic htt

**Fig 7:**
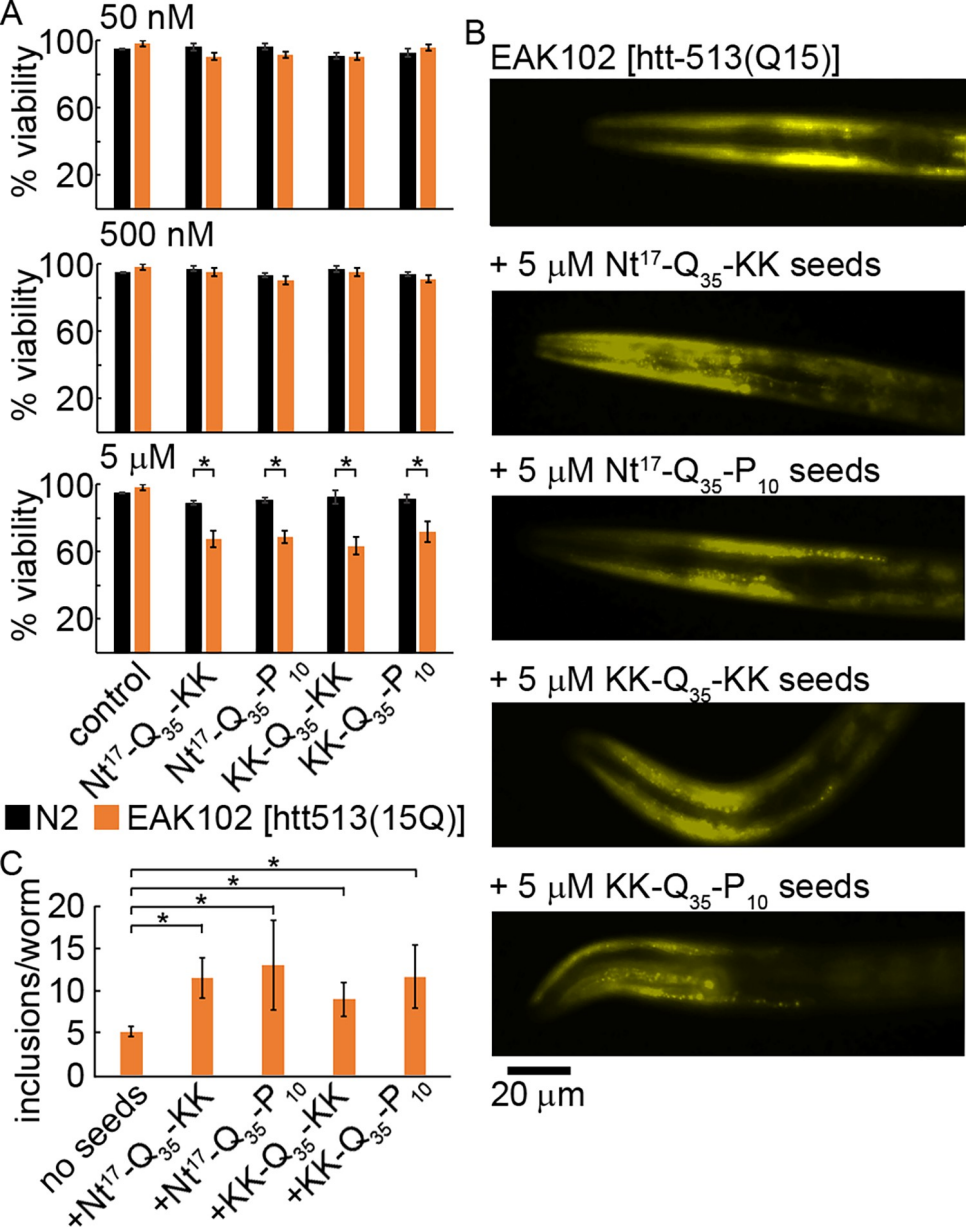
Peptide-derived fibrils seed aggregation in a *C*. *elegans* strain (EAK102) expressing a non-pathogenic N-terminal htt fragment. (A) The viability of N2 (not expressing htt) and EAK102 [expressing htt-513(Q15)] worms after 48 h of exposure to seeds derived from the peptides KK-Q_35_-KK, Nt^17^-Q_35_-KK, KK-Q_35_-P_10_-KK, and Nt^17^-Q_35_-P_10_-KK was determined. The control condition represents worms that were not exposed to any seeds. * indicates p < 0.05 based on a T-test (n = 3 for all conditions). Error bars represent the standard deviation. (B) Representative fluorescence microscopy images of an EAK102 worm that was not exposed to any peptide-derived seeds and EAK102 worms that were exposed to the various peptide-derived seeds (5 μM) for 48 h. (C) Quantification of the number of visual inclusion observed in EAK102 worms that have been exposed to peptide-derived seeds. * indicates p < 0.05 based on a T-test (n = 12 for no seeds and n = 5 for all other conditions). Error bars represent the standard deviation.

With all four htt peptide-derived fibrils being seeding competent, the ability of KK-Q_35_-KK, Nt^17^-Q_35_-KK, KK-Q_35_-P_10_-KK, and Nt^17^-Q_35_-P_10_-KK fibrils to seed aggregation in the EAK102 *C*. *elegans* strain expressing YFP-labeled, nonpathogenic htt-513(15Q) [43] was determined. Fibrils derived from all four peptides were sonicated to increase the efficiency of uptake and seeding. Control wild type N2 worms that do not express any htt fragments and the EAK102 worms were exposed to 50 nM, 500 nM, or 5 μM doses of the peptide-derived seeds on day 2 of adulthood. The viability of the worms was determined 48 h after exposure. For both N2 and EAK102 worms, there was no reduction in viability when not exposed to any seeds (Fig 7A control). Additionally, the 50 nM and 500 nM doses of peptide-derived seeds did not impact viability of either strain. With the 5 μM dose, the peptide-derived seeds were not toxic to N2 worms; however, all four peptide-derived seeds significantly (p < 0.05) reduced viability (~65–70%) of the EAK102 worms compared to N2 at this dose (Fig 7A). This suggests that, at least in part, toxicity derives from aggregation induced by the presence of polyQ seeds. While a small number of inclusions appeared in EAK102 worms that were not exposed to seeds, there was a significant (p <0.05) increase in the number of inclusions observed within worms exposed to the various peptide-derived seeds after 48 h (Fig 7B and 7C).

## Discussion

Induced htt aggregation by seeding plays a critical role in the cell to cell spread of HD [16–18]. Here, the ability of preformed htt-exon1 fibrils to seed aggregation *in vivo* was further demonstrated as the exposure of *C*. *elegans* expressing a non-pathogenic htt protein was sensitive to exposure to htt-exon1(46Q) seeds. This exposure resulted in reduced viability and invoked the formation of visible inclusions. To better understand the process by which htt fibrils seed aggregation, the role of polyQ length, flanking sequences, and membrane interfaces in modifying seeding of htt aggregation was investigated *in vitro*. Fibrils formed from pathogenic htt-exon1(46Q) proteins efficiently seeded aggregation of a non-pathogenic htt-exon1(20Q) protein. Peptides with a 35-repeat polyQ domain and varied combinations of flanking sequences seeded htt aggregation of both htt-exon1(46Q) and htt-exon1(20Q) with similar efficiencies, demonstrating that the polyQ fibril core plays the primary role in seeding. In all cases of seeding, the resulting fibril height depended on polyQ domain length of the targeted peptide. That is, seeded fibrils of htt-exon1(46Q) had the same morphology as fibrils formed from htt-exon1(46Q) without seeding, and seeded fibrils of htt-exon1(20Q) displayed smaller fibril heights compared with their htt-exon1(46Q) counterparts. Non-seeded fibrils of htt-exon1(20Q) were not observed. Fibrils of the model peptides formed in the presence of POPG vesicles all seeded aggregation of htt-exon1(46Q) with similar efficiencies. While the presence of TBLE vesicles suppressed fibril formation, htt peptide fibrils were still seeding competent in the presence of vesicles. However, fibril formation in the presence of TBLE vesicles never reached the level of fibril formation of htt in the absence of vesicles without seeds, suggesting that seeds only template free htt not bound to membranes and that membrane bound htt was at least partially protected from seeding. While not as robust in impact as htt-exon1(46Q) derived seeds, the synthetic peptide-derived seeds were selectively toxic to *C*. *elegans* expressing a nonpathogenic htt fragment in comparison to worms that do not express htt. Collectively, these results demonstrate that the polyQ domain within the fibril is the main driver of seeding efficiency and that htt proteins with nonpathogenic polyQ lengths are highly susceptible to seeded aggregation.

With regard to fibril formation from expanded polyQ domains, protein context and environment influence the process. Nt17 accelerates the process by facilitating the formation of α-helical rich oligomers that lower the barrier to nucleation by bringing polyQ domains into close proximity [47, 50]. The C-terminal polyP sequence slows aggregation [22, 23]. Despite the clear influence of flanking sequences in htt aggregation, the presence of these short flanking sequences does not appear to alter the seeding process. As seeding effectively bypasses fibril nucleation, this suggests that flanking sequences do not impact fibril elongation and that fibril growth is primarily associated with the polyQ domain. Based on X-ray diffraction patterns, the polyQ domain within fibrils consists of cross-β structure, a hallmark of amyloid. Additional structural analysis via circular dichroism (CD), Fourier-transform infrared spectroscopy (FTIR), and solid state NMR (ssNMR) validate that the fibril structure consists of antiparallel β-sheets polyQ core that is surrounded by the Nt17 and PRD flanking regions in a bottlebrush arrangement [21, 45, 46, 51–54]. In the fibril, the Nt17 domain remains in an immobilized α-helix attributable to density at the fibril surface. The PRD domain displays a restricted PPII-type helical structure for residues adjacent to the polyQ core due to packing density; however, further from the polyQ fibril core the PRD domain becomes unstructured [24, 55]. Importantly, the bottlebrush arrangement of these flanking sequences around the polyQ core do not block the ends of the fibril, maintaining solvent exposure of the polyQ core and creating active sites for fibril elongation. Our results suggest that the presence or absence of the flanking sequences does not alter the efficiency of fibril elongation and that fibrils formed from peptides with the same length polyQ domains have similar seeding efficiencies regardless of flanking sequences.

Interestingly, polymorphic fibril structure in htt-exon1 fibrils occurs within the PRD dependent on aggregation conditions, and these unique fibrils displayed varying degrees of seeding efficiency [27]. That is, fibrils with reduced entanglements in the PRD displayed exhibited enhanced seeding competency, and seeding correlated with enhanced cellular toxicity. While this appears to be in opposition to our observation that C-terminal polyP flanking sequences do not impact seeding competency of fibrils, it is important to note that the PRD domain of the complete htt-exon1 contains 50 residues past the polyQ domain, whereas, our synthetic peptides only had a 10 residue polyP flanking sequence. The larger bulk of the entire PRD may be providing steric hindrance, shielding the polyQ fibril core, and reducing seeding competency. With fewer entanglements [27], it is possible that the PRD is more easily penetrated in fibrils of full htt-exon1, allowing for access to the polyQ core. In our peptide system without the entire PRD, this phenomenon is not captured.

The presence of membranes further modifies htt aggregation in a lipid dependent manner [34, 35, 49, 56–58]. Flanking sequences also influence htt/lipid interactions and subsequent aggregation on the membrane surface. Nt17 plays a primary role by facilitating lipid binding via the formation of an amphipathic α-helix [32]. The influence of lipids on htt aggregation can be modified by post-translational modifications within Nt17 [59–62]. Here, four different polyQ peptides with differing combinations of flanking sequences were used to determine if flanking sequences altered the seeding efficiency of fibrils formed in the presence of lipids. Fibrils of all four peptides obtained by aggregation reactions in the presence of POPG vesicles, which enhance htt aggregation [34], displayed similar seeding efficiencies. This further supports the notion that the polyQ domain is a primary driver of seeding.

Another situation in which flanking sequence may alter seeding efficiency is when the seeding process occurs in the presence of lipids. One scenario is that seeds derived from different polyQ peptides with varying flanking sequences would display different affinities for membranes and be sequestered out of solution, altering their efficiency. This is not the case as polyQ-peptide derived seeds, regardless of flanking sequences, did not display any measurable affinity for lipid membranes based on PDA assays. In addition, the affinity of freshly prepared htt-exon1 for lipid membranes did not change with the addition of the different types of seeds in comparison to control experiments without seeds. This suggests two phenomena: 1) seeds do not alter the ability of htt monomers to bind membranes and 2) seeds do not impact the aggregation of htt bound to membranes. Based on ThT data, all four types of polyQ peptide fibrils seeded fibril formation in the presence of vesicles relative to htt aggregation reactions in the presence of vesicles without seeds. However, the steady state levels of fibril formation (based on maximum ThT signal) were always significantly smaller compared to htt aggregation without seeds in the absence of lipids. Collectively, this suggests that the polyQ-peptide derived seeds remain in solution and only seed aggregation of htt-exon1 not bound to lipids. The lipid-bound portion of htt-exon1 is protected and not available for addition to the growing fibrils present in solution. In effect, the presence of vesicles reduces htt-exon1 in solution, resulting in less fibril formation relative to aggregation in the absence of vesicles. Evidence for this scenario occurring in a cellular environment is provided by a recently developed assay for detecting seeding species in the cerebrospinal fluids of HD patients [18]. HEK-293 cell line expressing fluorescently labeled mutant htt(46Q), phosphomimetic mutant htt(46Q)-S13D/S16D, or mutant htt with a Nt17 deletion (ΔNt17) were exposed to htt-derived seeds, and formation of inclusion bodies was measured. Addition of mutant htt seeds to HEK-293 cells expressing mutant htt and the phosphomimetic htt resulted in a slight increase of inclusion formation. In contrast, the ΔNt17 construct was readily seeded, resulting in a several fold increase of inclusion formation relative to the other constructs. Similar to our *in vitro* experiments, the protein mutant htt and phosphomimetic mutant htt bound to lipids is protected from exogenous htt seeds, but deletion of Nt17 impairs the ability of htt to bind lipids, leaving the ΔNt17-htt available in the cytosol for seeding by exogenous htt aggregates.

Our study further supports the notion that fibrils derived from htt with expanded polyQ domains can seed aggregation of htt with nonpathogenic length polyQ domains. With seeding reactions involving mismatched polyQ domains, the resulting fibril morphology matches that of polyQ length being seeded, not that of the seed itself. This is likely due to the intrinsic polymorphism observed within the polyQ domain within fibrils. This ability to seed varying polyQ lengths may play a key role in HD onset. Disease progression and age of onset correlate with the speed of somatic expansion [63–65]. Somatic instability is the expansion of the CAG domain over time as a result of DNA damage and aberrant repair [63–65]. During the DNA repair of the CAG domain in the HTT gene, this trinucleotide-repeat folds over itself in a hairpin loop known as “slipped DNA.” This slipped DNA is incorporated into the gene sequences upon repair and with each erroneous slip the CAG repeat region continues to expand, enhancing aggregation and production of toxic aggregates [66, 67]. Cellular models demonstrate that altering certain DNA repair proteins prevents this somatic expansion. An example of this is the increased expression of FAN1 [67–69], a nuclease in the Fanconi anemia pathway. An increase in FAN1 slows progression of HD, results in later onset of HD, and shows increased stability of the CAG domain of the HTT gene [69]. Inversely, the loss-of-function variant of FAN1 (p.R507H) shows an earlier onset of HD. These aggregates can be internalized by healthy cells and seed aggregation of htt protein even when the gene has not undergone somatic expansion. In particular, the striatum and cortex of the brain appear to be hypermetabolic early into the disease and show a greater somatic instability from post-mortem brains [70].

Upon release from the native cell, seeding-competent htt aggregates are taken up by recipient cells, invoking aggregation in that cell and causing a progression of HD. Similar phenomenon occur in other neurodegenerative diseases such as Parkinson’s [71] and Alzheimer’s disease [72, 73]. Htt exits cells via a multitude of pathways, including synaptic transmission [74], vesicular transport [75], extracellular vesicles [76, 77], and tunneling nanotubes [19]. When mutant htt breaches cells in a lipid-free monomeric form, it can aggregate and move freely in the extracellular space, as demonstrated in N2A cells [17]. Cells also preferentially secrete mutant htt in comparison to wild type [78]. Lysosomal secretion allows cells to eliminate toxic htt species into the extracellular space; unfortunately, this release allows for mutant htt to invade adjacent cells. This prion-like dissemination and subsequent aggregation of htt throughout cells is observed in cellular and Drosophila models of HD [74, 75, 79]. For example, human neurons, upon integration with HD mouse brain slices, internalized mutant htt and exhibited aberrant morphology [76]. Here, *C*. *elegans* that do not express any htt were unaffected by exposure to htt-exon1 seeds; however, the viability of worms expressing htt with a nonpathogenic length polyQ domain viability was significantly reduced in a dose dependent manner. These worms also developed inclusions, demonstrating that seeding can play a key role in the development of HD.
